# Supplementary material for: Thioredoxin-1 distinctly promotes NF-κB target DNA binding and NLRP3 inflammasome activation independently of Txnip
Source: eLife. 2020 Feb 25;9:e53627. doi: 10.7554/eLife.53627 (PMC7062472; doi:10.7554/eLife.53627)
Supplement: Supplementary file 1. [file elife-53627-supp1.docx]

**Supplementary file 1. Sequences of primers used for RT-PCR.**

| **Reagent type (species) or resource** | **Designation** | **Source or reference** | **Identifiers** | **Additional information** |
| --- | --- | --- | --- | --- |
| Sequence-based reagent | *Arg1*_forward | This study | PCR primers | 5’- ACCTGGCCTTTGTTGATGTCCCTA-3’ |
| Sequence-based reagent | *Arg1*_reverse | This study | PCR primers | 5’- AGAGATGCTTCCAACTGCCAGACT-3’ |
| Sequence-based reagent | *Cd38*_forward | This study | PCR primers | 5’- GCCGGAGATGAGAGATCAG-3’ |
| Sequence-based reagent | *Cd38*_reverse | This study | PCR primers | 5’- GGGCGTAGTCTTCTCTTGTG-3’ |
| Sequence-based reagent | *Fizz1*_forward | This study | PCR primers | 5’- TCCAGCTGATGGTCCCAGTGAATA-3’ |
| Sequence-based reagent | *Fizz1*_reverse | This study | PCR primers | 5’- ACAAGCACACCCAGTAGCAGTCAT-3’ |
| Sequence-based reagent | *Gpr18*_forward | This study | PCR primers | 5’- GCACTCTCTCTCTGGGACTG-3’ |
| Sequence-based reagent | *Gpr18*_reverse | This study | PCR primers | 5’- TGGTATGTAGAACCTCCTGTCTG-3’ |
| Sequence-based reagent | *Grx1*_forward | This study | PCR primers | 5’- TGCAGAAAGACCCAAGAAATCCTCAGTCA-3’ |
| Sequence-based reagent | *Grx1*_reverse | This study | PCR primers | 5’- TGGAGATTAGATCACTGCATCCGCCTATG-3’ |
| Sequence-based reagent | *Grx2*_forward | This study | PCR primers | 5’- AAGGCTGTGGAGTTGGATATG-3’ |
| Sequence-based reagent | *Grx2*_reverse | This study | PCR primers | 5’- TATCCTGGGAACGGTTCTTTC-3’ |
| Sequence-based reagent | *Grx3*_forward | This study | PCR primers | 5’- ACGCTGTGGTTTCAGCAAG-3’ |
| Sequence-based reagent | *Grx3*_reverse | This study | PCR primers | 5’- GGATAGGTGGGCCAATTAGAG-3’ |
| Sequence-based reagent | *Grx5*_forward | This study | PCR primers | 5’- GAGCTGAGGCAAGGTATTAAAG-3’ |
| Sequence-based reagent | *Grx5*_reverse | This study | PCR primers | 5’- CTCGCCGTTGAGGTACAC-3’ |
| Sequence-based reagent | *Hif1a_*forward | This study | PCR primers | 5’- AGCTTCTGTTATGAGGCTCACC-3’ |
| Sequence-based reagent | *Hif1a*_reverse | This study | PCR primers | 5’- TGACTTGATGTTCATCGTCCTC-3’ |
| Sequence-based reagent | *Il12b*_forward | This study | PCR primers | 5’- TCATCAGGGACATCATCAAAC-3’ |
| Sequence-based reagent | *Il12b*_reverse | This study | PCR primers | 5’- TTGAGGGAGAAGTAGGAATGG-3’ |
| Sequence-based reagent | *Il1b_*forward | This study | PCR primers | 5’- CCACCTTTTGACAGTGATGAG-3’ |
| Sequence-based reagent | *Il1b*_reverse | This study | PCR primers | 5’- CCAGGTCAAAGGTTTGGAAGC-3’ |
| Sequence-based reagent | *Il6*_forward | This study | PCR primers | 5’- TTCCATCCAGTTGCCTTCTTG-3’ |
| Sequence-based reagent | *Il6*_reverse | This study | PCR primers | 5’- TCATTTCCACGATTTCCCAGA-3’ |
| Sequence-based reagent | *Nos2*_forward | This study | PCR primers | 5’- CCAAGCCCTCACCTACTTCC-3’ |
| Sequence-based reagent | *Nos2*_reverse | This study | PCR primers | 5’- CTCTGAGGGCTGACACAAGG-3’ |
| Sequence-based reagent | *Txn1*_forward | This study | PCR primers | 5’- ATGACTGCCAGGATGTTGC-3’ |
| Sequence-based reagent | *Txn1*_reverse | This study | PCR primers | 5’- CCTTGTTAGCACCGGAGAAC-3’ |
| Sequence-based reagent | *Txnip*_forward | This study | PCR primers | 5’- CCTGACCTAATGGCACCAG-3’ |
| Sequence-based reagent | *Txnip*_reverse | This study | PCR primers | 5’- AGGAATGAACATGCAGGAAAC-3’ |
| Sequence-based reagent | *Txnip* mRNA in KO_forward | This study | PCR primers | 5’- CTTCACCCCCCTAGAGTGAT-3’ |
| Sequence-based reagent | *Txnip* mRNA in KO_reverse | This study | PCR primers | 5’- CCCAGAGCACTTTCTTGGAC-3’ |
| Sequence-based reagent | *Txnrd1*_forward | This study | PCR primers | 5’- AAAGACGATGAACGTGTCG-3’ |
| Sequence-based reagent | *Txnrd1*_reverse | This study | PCR primers | 5’- CTTAGTCAGCCCACACTTGAG-3’ |
| Sequence-based reagent | *Txnrd1* gDNA_forward | This study | PCR primers | 5’- ACAGGAGTGATCCCCACAGACC-3’ |
| Sequence-based reagent | *Txnrd1* gDNA_reverse | This study | PCR primers | 5’- CTGGAACCGCCCTGAATATCACC-3’ |
| Sequence-based reagent | *Txnrd1* mRNA in KO_forward | This study | PCR primers | 5’- GCTGACTAAGCAGCAGCTGG-3’ |
| Sequence-based reagent | *Txnrd1* mRNA in KO_reverse | This study | PCR primers | 5’- AACCTCAGCAGCCAGACTGG-3’ |
| Sequence-based reagent | *Ym1*_forward | This study | PCR primers | 5’- AGAAGGGAGTTTCAAACCT-3’ |
| Sequence-based reagent | *Ym1*_reverse | This study | PCR primers | 5’- GTCTTGCTCATGTGTGTAAGTGA-3’ |
| Sequence-based reagent | *Tbp*_forward  (housekeeping for mRNA) | This study | PCR primers | 5’- TTGACCTAAAGACCATTGCACTTC-3’ |
| Sequence-based reagent | *Tbp*_reverse  (housekeeping for mRNA) | This study | PCR primers | 5’- TTCTCATGATGACTGCAGCAAA-3’ |
| Sequence-based reagent | *Txnrd1*_forward  (housekeeping for DNA) | This study | PCR primers | 5’- ACAGATCGAAGCAGGAACAC-3’ |
| Sequence-based reagent | *Txnrd1*_reverse  (housekeeping for DNA) | This study | PCR primers | 5’- TTCAGAGAGGAAAGTCACCC-3’ |
| Sequence-based reagent | *Il12b*  *“P*romoter”_forward  (for ChIP) | This study | PCR primers | 5’- AAGCACCAGGAGCAGCCAA-3’ |
| Sequence-based reagent | *Il12b*  *“P*romoter”_reverse  (for ChIP) | This study | PCR primers | 5’- CTGGAGTCTGAATCTGGAC-3’ |
| Sequence-based reagent | *Il12b “*Ctr primers”_forward  (for ChIP) | This study | PCR primers | 5’- GGAAGCACGGCAGCAGAA-3’ |
| Sequence-based reagent | *Il12b “*Ctr primers”_reverse  (for ChIP) | This study | PCR primers | 5’- ATCATGGACACGTGGCAG-3’ |
| Sequence-based reagent | *Il1b “P*romoter”_forward  (for ChIP) | This study | PCR primers | 5’- GGTAGCAATAGCCTCTTCC-3’ |
| Sequence-based reagent | *Il1b “P*romoter”_reverse  (for ChIP) | This study | PCR primers | 5’- CAGGGTTTGTTGTCCAACTTG-3’ |
| Sequence-based reagent | *Il1b “*Ctr primers”_forward  (for ChIP) | This study | PCR primers | 5’- CCATGAGCTTTGTACAAGG-3’ |
| Sequence-based reagent | *Il1b “*Ctr primers”_reverse  (for ChIP) | This study | PCR primers | 5’- GGCTTCTCTACTGATGGA-3’ |
| Sequence-based reagent | *Il6 “P*romoter”_forward  (for ChIP) | This study | PCR primers | 5’- AATCAGCCCCACCCACTC-3’ |
| Sequence-based reagent | *Il6 “P*romoter”_reverse  (for ChIP) | This study | PCR primers | 5’- GCTCCAGAGCAGAATGAG-3’ |
| Sequence-based reagent | *Il6 “*Ctr primers”_forward  (for ChIP) | This study | PCR primers | 5’- CCTCTGGTCTTCTGGAGT-3’ |
| Sequence-based reagent | *Il6 “*Ctr primers”_reverse  (for ChIP) | This study | PCR primers | 5’- TCCCACACTGCATGAGAG-3’ |
| Sequence-based reagent | *Nfkbia “P*romoter”_forward  (for ChIP) | This study | PCR primers | 5’- AGGCTGCAGGGAAGTACCT-3’ |
| Sequence-based reagent | *Nfkbia “P*romoter”_reverse  (for ChIP) | This study | PCR primers | 5’- TTTCCAAGCCAGTCAGACT-3’ |
| Sequence-based reagent | *Nfkbia “*Ctr primers”_forward  (for ChIP) | This study | PCR primers | 5’- GTGCTGATGTCAACGCTC-3’ |
| Sequence-based reagent | *Nfkbia “*Ctr primers”_reverse  (for ChIP) | This study | PCR primers | 5’- CAACAAGAGCGAAACCAG-3’ |
